# Correspondence of directly reported and recalled health-related quality of life in a large heterogeneous sample of trauma patients

**DOI:** 10.1007/s11136-019-02256-z

**Published:** 2019-07-30

**Authors:** I. Spronk, A. J. L. M. Geraerds, G. J. Bonsel, M. A. C. de Jongh, S. Polinder, J. A. Haagsma

**Affiliations:** 1 Department of Public Health, Erasmus MC, University Medical Center Rotterdam, P.O. Box 2040, 3000 CA Rotterdam, The Netherlands; 2 Association of Dutch Burn Centres, Maasstad Hospital, Rotterdam, The Netherlands; 3 Department of Plastic, Reconstructive and Hand Surgery, Amsterdam Movement Sciences, Amsterdam UMC, Vrije Universiteit Amsterdam, Amsterdam, The Netherlands; 4 Division Mother and Child, Utrecht University Medical Center, Utrecht, The Netherlands; 5 Department Trauma TopCare, ETZ Hospital, Hilvarenbeekseweg 60, 5022 GC Tilburg, The Netherlands

**Keywords:** Health-related quality of life, EQ-5D, Retrospective assessment, Trauma population

## Abstract

**Purpose:**

To evaluate the correspondence of directly reported and recalled health-related quality of life (HRQL) in a heterogeneous sample of trauma patients.

**Methods:**

Adult trauma patients who attended the Emergency Department and were admitted between 03/2016 and 11/2016 were invited to participate. Postal surveys were sent 1 week (T1), 3 months (T2), and 12 months (T3) post-trauma. The EQ-5D-3L and Visual Analogue Scale (EQ-VAS) were used to assess directly reported and recalled HRQL.

**Results:**

The EQ-5D was completed by 446 patients at T1, T2, and T3. Directly reported mean T1 EQ-5D summary score was 0.482, whereas recalled T1 EQ-5D summary score was 0.453 (*p* < 0.05) at T2 and 0.363 (*p* < 0.001) at T3. Directly reported mean T2 EQ-5D summary score was 0.737 and mean recalled T2 EQ-5D summary score was 0.713 (*p* < 0.05) at T3. Directly reported mean T1 EQ-VAS was 56.3, whereas mean recalled T1 EQ-VAS at T2 and T3 was 55.4 (*p* = 0.304) and 53.3 (*p* < 0.05), respectively. Directly reported mean T2 EQ-VAS was 72.5 and recalled T2 EQ-VAS at T3 was 68.0 (*p* < 0.001). The correspondence between all directly reported and recalled HRQL (both EQ-5D summary and EQ-VAS) was fair (ICC = 0.518–0.598). Lowest correspondence was seen in patients with major trauma (injury severity score ≥ 16) and in patients with middle-level education.

**Conclusions:**

Recalled HRQL measured by the EQ-5D-3L and EQ-VAS was systematically lower compared to the directly reported HRQL. Patient characteristics, injury severity, subjectivity of the dimension, and time interval appear to influence correspondence between directly reported and recalled HRQL.

**Electronic supplementary material:**

The online version of this article (10.1007/s11136-019-02256-z) contains supplementary material, which is available to authorized users.

## Introduction

An important outcome in trauma care is health-related quality of life (HRQL) of patients. HRQL reflects a patient’s physical, psychological, and social well-being [1]. This subjective measurement is increasingly used in estimating the impact of an injury, in evaluating the quality of care provided, and in providing patient information on particular injuries [2, 3]. Measurement of HRQL changes over time may be additionally useful to understand patterns of recovery over time and the role of rehabilitative care [4, 5].

It is, however, a challenge to establish reliable and valid outcomes for changes of HRQL over time. The best time frame to measure relevant changes over time may be difficult to define ex ante, data may be incomplete due to censoring (death, withdrawal) or random missings, and the event itself may be unpredictable, which makes prospectively collecting HRQL data difficult or impossible [4]. Retrospective assessment can be used to reconstruct the HRQL at an earlier time point. Retrospective assessment is easier to implement and involves less patient burden, but may be confounded by recall bias [6], and response shift may occur [7–9]. Recall bias is defined as a systematic measurement error, due to memory decay, which is the fading of memory with time. As a result, patients may remember their HRQL as being better or worse than it actually was [10]. Response shift on the other hand is the change in the meaning of a person’s evaluation of a specific construct. This can be caused by a change in internal standards, a change in values, and/or a redefinition of the construct [11, 12]. Among trauma patients, response shift may occur between multiple post-injury HRQL measurements due to patients adapting to their ill health.

Conventionally measured change in HRQL (post-level minus pre-level) may not be identical to the change in HRQL as reported by the patient, looking back at the time point of interest (retrospective change). If we take post-level minus pre-level as gold standard, retrospective recall bias will depend on the time interval between the measurement and the recall moment, as bias likely increases with longer intervals between measurements [6]. The presence of recall effects may also depend on the scale used, where a visual analogue scale (VAS) with a wide range of response options may be easier distorted than a classification-like scale with a limited number of response options, like the descriptive system of the EQ-5D-3L [13]. Finally, adequate props and instructions may support retrospective measurement avoiding the tendency to create emotionally fitting stories (cognitive dissonance reduction) [14].

Only few studies with varying results have evaluated the correspondence of patient recall of HRQL. Correspondence was poor [intraclass correlation coefficient (ICC) 0.34–0.40] among a sample of elderly hospitalized patients (3 day vs. 38 days assessment). A large proportion of this poor correspondence was attributed to recall bias; the correspondence after adjustment for recall bias was excellent (ICC 0.90–0.98) [15]. Two other studies in patients with prostate cancer found moderate correspondence (ICC 0.39–0.57) between pre-surgery HRQL and recalled pre-surgery HRQL (pre-surgery and 6–37 months post-surgery assessment) [16, 17], and a study in patients with hip arthroplasty found good correspondence of pre-surgery HRQL and retrospectively assessed HRQL at various time points (3 days, 6 weeks, and 3 months assessment) post-surgery (ICC 0.70–0.95) [18].

This study is the first study ever to evaluate the correspondence of directly reported HRQL and recalled HQRL in a heterogeneous sample of trauma patients, with specific attention to predefined subgroups. It compares directly reported EQ-5D summary and EQ-VAS scores collected at 1 week and 3 months post-injury, and recalled scores of 1 week collected at 3 months and 12 months post-injury, and recalled scores of 3 months collected at 12 months.

## Methods

### Study design

The present study is part of the Brabant Injury Outcome Surveillance (BIOS) study. This prospective longitudinal cohort study assesses HRQL in trauma patients who were admitted to one of 10 hospitals in the region Noord-Brabant (the Netherlands) [19]. The follow-up period in this dataset was 24 months; however, recall questions were only included at the 3 months and 12 months survey. Therefore, the 12-month follow-up data were used for the present study. Approval for the BIOS study was given by the Medical Ethics Committee Brabant (NL50258.028.14).

### Participants

Participants were adult (≥ 18 years) trauma patients, with an intake at the Emergency Department (ED) and who were admitted to either an Intensive Care unit (ICU) or a ward of one of the ten hospitals between March 2016 and November 2016. Only patients who survived hospital discharge were included. Patients who were unable to reply to Dutch language questionnaires, patients with a pathological fracture due to a malignancy or metastasis, and patients without a permanent address were excluded [19]. 1 week after their hospital admission, all eligible patients were invited to participate in the present study via a postal invitation, including an informed consent form and the first questionnaire (T1). Non-responders received a phone call to discuss participation one week after receiving the questionnaire. After consent was given, subsequent recall questionnaires were sent 3 months (T2), and 12 months (T3) post-trauma. Only data from patients who completed all items in all questionnaires were included in the analysis. Informed consent was obtained from all individual participants in the study.

### Measures

The first questionnaire covered patient characteristics, like age and gender, and the presence of self-reported chronic morbidity, e.g., diabetes. In such cases the patient was defined as having comorbidity [20]. All questionnaires included the EQ-5D-3L, which is a preference-based measure to estimate utility that was used to assess patients health status. It includes five dimensions: mobility, self-care, usual activities, pain/discomfort, and anxiety/depression. The five dimensions have three ordered response options: no problems, moderate problems, and extreme problems [21]. Based on these five dimensions, a summary score (through weighting) was calculated by using the Dutch value set. The summary score can range between 0 (death) and 1 (full health) [22]. The summary score rarely has a negative value for health states stated to be worse than death. Besides, the EQ-5D-3L includes a visual analogue scale (EQ-VAS) [23], consisting of a scale from 0 (worst imaginable health) to 100 (best imaginable health). Participants were asked to complete the EQ-5D-3L and the EQ-VAS for the current situation in all questionnaires. At T2 and T3 they were also asked to report what they remember to have reported on the EQ-5D-3L at the previous assessment(s). We added a general statement emphasizing the recalled time point of interest (T1 or T2) to each of the EQ-5D-3L questionnaires. At T3, we first asked participants to recall T1 and then T2. At T2, the T1 recall was asked and at T3, the T2 and T1 recall was requested.

### Injury data

Injury data of included patients were available from the Brabant Trauma Registry. In this registry, all ten participating hospital are included. Data included the Abbreviated Injury Scale (AIS) [24]. The AIS classifies the severity of a trauma via an anatomic reference scale. The AIS describes type, location, and rates—the severity in numbers. Based on the highest AIS score in each injured body region, the Injury Severity Score (ISS) is composed as follows. The three most severely injured body regions according to the severity rating are selected, and the severity scores are squared and summed up. By definition the ISS ranges from 1 to 75. A major trauma is defined as an ISS ≥ 16 [25].

### Hypotheses

Our hypotheses are:

- Correspondence between the directly reported and recalled score is lower with the EQ-VAS compared to the EQ-5D summary score as the EQ-5D descriptive system has a limited number of response options and is thus expected to be less prone to recall bias.
- Correspondence between the directly reported and recalled score is higher with the 3-month window compared to the 9- and 12-month window as bias likely increases with longer intervals between measurements
- Correspondence between the directly reported and recalled score is lower among severely injured patients (ISS ≥ 16) as we expect a stronger cognitive dissonance among these patients because their rehabilitation period is long and patients adapt to their non-optimal post-state.

### Statistical analysis

All analyses were performed using SPSS version 23. A non-response analysis was performed to test for differences among responders and non-responders. Chi-square tests were used for categorical variables and Mann–Whitney U tests for continuous variables. We compared T1–T2, T1–T3, and T2–T3 correspondence of direct (i.e., the EQ-5D outcome at that moment) versus recalled outcomes for the EQ-5D summary, the dimensions, and the EQ-VAS scores. The paired *t* test was used to compare the direct versus recalled outcomes for all participants, and for subgroups based on age, gender, education, comorbidity, and ISS. For the subgroup paired *t* test, we split the sample into the aforementioned subgroups. Additionally, we used the intraclass correlation coefficient (ICC) [26]. The ICC describes quantitative correspondence of two numericals. Again this analysis was done to compare direct versus recalled outcomes on an individual level for all patients and the subgroups. ICC was defined as being poor (< 0.40), fair (0.40–0.59), good (0.60–0.74), or excellent (0.75–1.00) [27]. A perfect ICC (1.0) is the result of no difference on the individual level. However, an imperfect ICC (say 0.6) may point to a systematic difference between direct and recall outcomes [e.g., recall always ‘better’] which in turn will lead to a significant difference in *t* test terms (provided numbers are reasonable), or it may point to a random difference between the two [e.g., due to vague memory] which in turn will lead to no (group) difference in *t* test terms. Hence, recall bias and error both limit the ICC, but only bias affects the *t* test.

## Results

### Participants

In total, 5731 trauma patients were invited to participate in present study, of whom 1518 patients (26.5%) agreed to participate. The questionnaire within 1 week of the trauma (T1) was completed by 759 participants (50.0%), the questionnaire at 3 months (T2) by 1294 participants (85.2%) and at 12 months (T3) by 1255 participants (82.7%). In total, 551 participants returned the three questionnaires and the direct EQ-5D and recall EQ-5D were completed by 446 participants (29.4%) for T1–T2–T3. Non-response analysis showed that participants were significantly younger (*p* < 0.05) and more often males (*p* < 0.05) than non-respondents.

Responders had a mean age of 61.5 years (SD 15.3) and 55% was male (Table 1). Many responders had middle or high level education and comorbidity was highly prevalent; more than half (57%) of the patients had a chronic disease. Median hospital stay was 4.0 days (IQR 2.0–6.0 days). The most common injuries were mild traumatic brain injury (28%) and hip fracture (21%). Median ISS was 5.0 (IQR 4.0–9.0), with 29 participants (7%) having a major trauma.

**Table 1:** Characteristics of study population

Characteristic	Participants (*n* = 446)
Gender: Male	247 (55.4%)
Age (M, SD)	61.5 (15.3)
Education	
Low	97 (21.7%)
Middle	171 (38.3%)
High	171 (38.3%)
Unknown	7 (1.6%)
Comorbidity status	
No comorbidity	185 (41.5%)
Comorbidity	253 (56.7%)
Unknown	8 (1.8%)
Length of hospital stay (Median, IQR)	4.0 (2.0-6.0)
Injury type	
Pelvic injury	56 (12.6%)
Hip fracture	93 (20.9%)
Tibia, complex foot or femur fracture	58 (13.0%)
Shoulder and upper arm injury	53 (11.9%)
Radius, ulna or hand fracture	32 (7.2%)
Mild TBI	124 (27.8%)
Severe TBI	10 (2.2%)
Facial fracture	23 (5.2%)
Thoracic injury	25 (5.6%)
Rib fracture	60 (13.5%)
Mild abdominal injury	10 (2.2%)
Severe abdominal injury	3 (0.7%)
Spinal cord injury	2 (0.4%)
Stable vertebral fracture or disc injury	31 (7.0%)
Injury severity score	
< 8	255 (57.2%)
8–16	162 (36.3%)
≥ 16	29 (6.5%)

*SD* standard deviation, *IRQ* inter quartile range

### EQ-5D summary scores

#### Directly reported versus recalled measurement comparisons

**Fig. 1:**
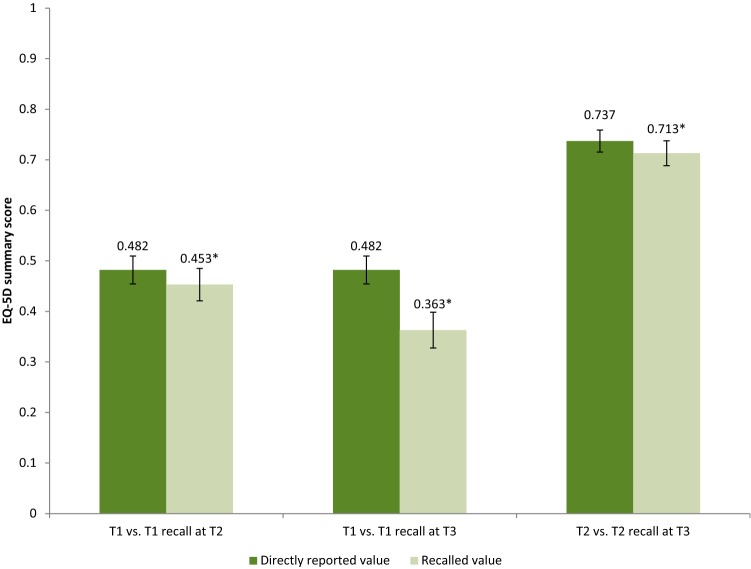
Mean and confidence interval EQ-5D summary score assessed 1 week (T1) and recall at 3 months (T2: recall T1) and 12 months (T3: recall T1) post-injury; and mean EQ-5D summary score assessed 3 months (T2) and recall at 12 months (T3: recall T2) post-injury. *Statistically significant (*p *< 0.05)

The directly reported mean T1 EQ-5D summary score was 0.482, whereas the recalled T1 EQ-5D summary was 0.453 (*p* < 0.05) at T2 and 0.363 (*p* < 0.001) at T3 (Fig. 1). The directly reported mean T2 EQ-5D summary score was 0.739 and the recalled T2 EQ-5D summary score was 0.713 (*p* = 0<0.05) at T3 (Fig. 1). Confidence intervals of the recalled scores were larger than the direct scores. The proportion of respondents that reported exact the same, lower, and higher scores are displayed in Table 2. Absolute individual differences in EQ-5D summary scores between T1 and recalled T1 at T2 ranged from − 0.97 to 1.14; differences between T1 and recalled T1 at T3 ranged from − 1.13 to 1.20; and differences between T2 and recalled T2 at T3 ranged from − 1.13 to 1.33. The recalled EQ-5D summary scores were lower compared to the direct scores in most studied subgroups, except for the group with a low educational level for T2 versus recalled T2 at T3 (Online Resource 1–3). Some of these differences were statistically significant. The differences between the directly reported T1 score and the recalled T1 score at T2 were significantly different in the subgroup of females, the subgroup < 65 years old, and the subgroup with a middle education level. For the recalled T2 score at T3 differences were significant in the subgroup females, the subgroup < 65 years old, the subgroup with a middle educational level, the subgroup with comorbidity, and the subgroup with major injury (ISS ≥ 16). The differences between T1 and the recalled T1 at T3 were significantly different for all subgroups.

**Table 2:** Correspondence of directly reported and recalled EQ-5D summary and EQ-VAS assessed 1 week (T1), 3 months (T2), and 12 months (T3) post-injury

	Directly reported = recalled (%)	Directly reported < recalled (%)	Directly reported > recalled (%)
T1–T2 recall			
EQ-5D summary	22.4	36.5	41.0
EQ-VAS	4.0	47.8	48.2
T1–T3 recall			
EQ-5D summary	16.4	28.7	54.9
EQ-VAS	5.6	40.4	54.0
T2–T3 recall			
EQ-5D summary	30.5	30.7	38.8
EQ-VAS	5.8	30.9	63.2

Directly reported = recalled: respondents filled in exactly the same EQ-5D and EQ-VAS answers for the recall

Directly reported < recalled: respondents reported higher scores (less problems) when EQ-5D and EQ-VAS were recalled

Directly reported > recalled: respondents reported lower scores (more problems) when EQ-5D and EQ-VAS were recalled

#### Correspondence directly reported versus recalled scores

The correspondence was fair (0.40–0.59) for all comparisons (Online Resource 1–3). This was also shown by the Bland–Altman plots (Online Resource 4–6). The ICC was worst for the recalled T1 score at T3 (ICC = 0.518) and best for the recalled T2 score at T3 (ICC = 0.598). Within the subgroups, the correspondence was lowest in the subgroup < 65 years old (ICC = 0.498) on T1 versus recalled T1 at T2, and the subgroup with a middle education on T1 versus recalled T1 at T3 (ICC = 0.423) and T2 versus recalled T2 at T3 (ICC = 0.483). For T1 versus recalled T1 at T2 the correspondence was highest in the subgroup ≥ 65 years old (ICC = 0.647), for T1 versus recalled T1 at T3 in the subgroup with a low education level (ICC = 0.627), and for T2 versus recalled T2 at T3 in the subgroup with a high educational level (ICC = 0.673).

### EQ-5D dimensions

Directly reported and recalled dimension scores were also compared. The recalled scores for the dimension anxiety were significantly different for all three comparisons (all *p *< 0.05) (Table 3). Furthermore, the score for the dimension daily activities was significantly different from its direct score on T1 versus recalled T1 at T2 (*p *< 0.05), and the score for the dimension self-care on T2 versus recalled T2 at T3 (*p *< 0.05). And all dimension scores, except for daily activity (*p *= 0.197), were significantly different (*p *< 0.001) on T1 versus recalled T1 at T3. The correspondence was lowest for the dimension anxiety/depression for T1 versus recalled T1 at T2 (ICC = 0.444) and for T1 versus recalled T1 at T3 (ICC = 0.371) and pain/discomfort for T2 versus recalled T2 at T3 (ICC = 0.484). The correspondence was best for mobility on all comparisons (ICC 0.642–0.676).

**Table 3:** EQ-5D dimension score assessed at 1 week (T1) and recall at 3 months (T2) and at 12 months (T3) post-injury and intraclass correlation coefficients (ICC) (*n* = 446)

EQ-5D dimension	*p* value	ICC (95% CI)
T1–T2 recall		
Mobility	0.244	0.676 (0.62, 0.72)
Self-care	0.757	0.632 (0.57, 0.68)
Daily activities	0.019*	0.520 (0.45, 0.58)
Pain/discomfort	0.186	0.474 (0.40, 0.54)
Anxiety/depression	< 0.001*	0.444 (0.37, 0.52)
EQ-5D summary score	0.044*	0.575 (0.51, 0.63)
EQ-VAS	0.304	0.578 (0.51, 0.64)
T1–T3 recall		
Mobility	< 0.001*	0.642 (0.58, 0.69)
Self-care	< 0.001*	0.567 (0.50, 0.63)
Daily activities	0.197	0.510 (0.44, 0.58)
Pain/discomfort	< 0.001*	0.383 (0.30, 0.46)
Anxiety/depression	< 0.001*	0.371 (0.29, 0.45)
EQ-5D summary score	<0.001*	0.518 (0.45, 0.58)
EQ-VAS	0.002*	0.561 (0.49, 0.62)
T2–T3 recall		
Mobility	0.831	0.655 (0.60, 0.71)
Self-care	0.004*	0.513 (0.44, 0.58)
Daily activities	0.766	0.554 (0.49, 0.62)
Pain/discomfort	0.511	0.484 (0.41, 0.55)
Anxiety/depression	0.031*	0.523 (0.45, 0.59)
EQ-5D summary score	0.022*	0.598 (0.54, 0.65)
EQ-VAS	< 0.001*	0.595 (0.53, 0.65)

*ICC* Intraclass correlation coefficient

*p < 0.05

### EQ-VAS

#### Directly reported versus recalled measurement comparisons

**Fig. 2:**
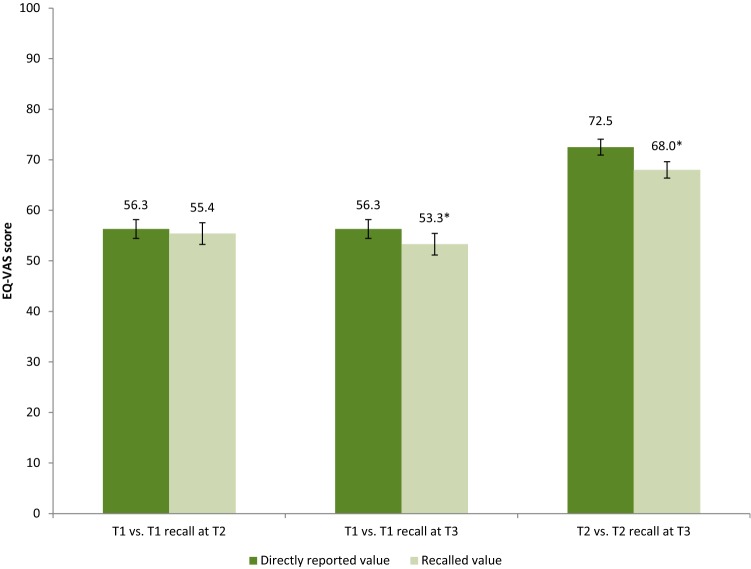
Mean and confidence interval EQ-VAS score assessed 1 week (T1) and recall at 3 months (T2: recall T1) and 12 months (T3: recall T1) post-injury; and mean EQ-VAS score assessed 3 months (T2) and recall at 12 months (T3: recall T2) post-injury. *Statistically significant (*p *< 0.05)

The directly reported mean T1 EQ-VAS score was 56.3, the recalled T1 EQ-VAS score was 55.4 (*p *= 0.304) at T2 and 53.3 (*p *< 0.05) at T3 (Fig. 2). The directly reported mean T2 EQ-VAS score was 72.5 and the recalled T2 score was 68.0 (*p *< 0.001) at T3 (Fig. 2). Confidence intervals of the recalled scores were larger than of the directly reported scores. The proportion of respondents that reported the exact same, lower and higher scores are displayed in Table 2. Absolute individual differences in EQ-VAS scores between T1 and recalled T1 at T2 ranged between − 99 and 57; differences between T1 and recalled T1 at T3 ranged between − 100 and 70; and differences between T2 and recalled T2 at T3 ranged between − 76 and 90. Recalled EQ-VAS scores were lower than the directly reported EQ-VAS scores, except for the subgroup 65 + for T1 versus recalled T1 at T2 and for T1 versus recalled T1 at T3, and the subgroup with low education on T2 versus recalled T2 at T3. Subgroup results are presented in Online Resource 7–9. Comparing the recalled T1 at T2 with the directly reported T1 resulted in no statistical differences, except for the subgrou*p *< 65 years old (*p *< 0.05) and the subgroup with middle level of education (*p *< 0.05), while the directly reported T2 EQ-VAS and recalled T2 EQ-VAS at T3 was statistically significant different for all subgroups, except for the subgroup with low level of education. For the recalled T1 EQ-VAS at T3, about half of the subgroups showed statistical significant differences between the directly reported and the recalled EQ-VAS (Online Resource 8).

#### Correspondence directly reported versus recalled scores

The correspondence between directly reported and recalled EQ-VAS scores was fair on all time points (ICC 0.561–0.595) (Online Resource 10–12). The correspondence was lowest for T1 versus recalled T1 at T3 (ICC = 0.561), slightly better for T1 versus recalled T1 at T2 (ICC = 0.578) and highest for T2 versus recalled T2 at T3 (ICC = 0.595). Within the subgroups, the correspondence was lowest in the subgroup with a high ISS for T1 versus recalled T1 at T2 (ICC = 0.188) and T1 versus recalled T1 at T3 (ICC = 0.292) and in the subgroup with a middle educational level for T2 versus recalledT2 at T3 (ICC = 0.512), resembling EQ-5D summary score results. The correspondence was best in the subgroup females for T1 versus recalled T1 at T2 (ICC = 0.636), and in the subgroup with a high educational level for T1 versus recalled T1 at T3 (ICC = 0.652) and T2 versus recalled T2 at T3 (ICC = 0.703).

## Discussion

This study explored the recall effects of HRQL assessment in a large heterogeneous sample of trauma patients. The results showed that recalled HRQL measured by the EQ-5D-3L and EQ-VAS is systematically lower compared to the directly reported HRQL of trauma patients, with a general decrease over time. The relative size of measurement error and bias was larger in EQ-5D-3L summary scores than in EQ-VAS. Most distortion in recalled HRQL was present in the dimensions anxiety/depression and pain/discomfort. The correspondence between directly reported and recalled scores decreased with the time between measurements, and it was influenced by the post-injury phase being recalled: correspondence was better when T2 (3-months post-injury; recovery phase) was recalled compared to when T1 (1-week post-injury; acute phase) was recalled. Patients with a major injury and those with a middle level of education had most difficulties with recalling their prior HRQL, whereas patients with a high educational level were in general best in recalling their prior HRQL.

Our study showed in general fair correspondence between directly reported and recalled HRQL. This is in line with earlier studies on HRQL recall that showed that the association between recalled HRQL and prospective reports of HRQL was moderate [13]. This was the case in patients with prostate cancer [16, 17] as well as in older hospital patients [15]. Two studies on recall of pre-surgery HRQL in prostate cancer found correlations between 0.39 and 0.57 for scores collected before and six to 37 months after surgery [16, 17]. In the study of McPhail et al., elderly hospitalized patients reported their HRQL within 3 days of admission and immediately prior to discharge (median hospital stay of 38 days). This study found a poor recall correspondence (ICC of 0.34 for EQ-5D summary score and 0.40 for EQ-VAS) [15]. However, as opposed to the results of these studies, a study in patients with hip arthroplasty found good to excellent correspondence of pre-surgery HRQL scores obtained before surgery and 3 days (ICC 0.8–0.9), 6 weeks (ICC 0.7–0.9), and 3 months (ICC 0.85–0.95) post-surgery [18]. Results on recall correspondence are thus scarce and seem to depend on the condition that is being recalled as well as on the time frame between the assessments. Earlier studies investigated the test–retest reliability of the EQ-5D-3L. These studies showed that the accuracy of the EQ-5D-3L differed, depending on the timeframe, EQ-5D-3L utility or VAS used, and study population and ranged from 0.70 to 0.85 [28–31]. The correspondence between directly reported and recalled HRQL based on the EQ-5D-3L found in our study is much lower, as we expected, since correspondence between directly reported and recalled HRQL cannot be more accurate than the reliability of the instrument. However, it should be noted that test–retest reliability of the EQ-5D-3L was not yet studied in trauma patients and therefore we were not able to compare the correspondence found against the accuracy of the instrument in trauma patients.

As opposed to our hypothesis that a scale with a wider range of response options like the EQ-VAS is easier distorted than a classification-like scale with a limited number of response options, like the EQ-5D-3L [13], our findings showed lower ICC scores on the EQ-5D-3L compared to the EQ-VAS. This was also seen in the study of McPhail et al. where the ICC score of the EQ-VAS was higher than the score of the EQ-5D summary (0.40 vs. 0.34) [15]. In view of these results, we reject our hypothesis as the EQ-VAS seems to be less distorted compared to the EQ-5D-3L.

Also, the time interval between the initial measurement and the recall moment was seen to influence the correspondence of recall; however, results were partly in contrast with our hypothesis. As expected, recalled scores of 1 week post-trauma differed more from the directly reported scores when recalled at 12 months post-injury compared to 3 months post-injury. This is in line with earlier studies that showed that the correspondence of recall decreases with the time between the initial measurement and the recalled moment [10]. However, despite the longer time of 9 months between the initial assessment at 3 months and the recall assessment at 12 months, the correspondence between T2 and T3 was higher (highest ICC rates) compared to the T1 and T2. This seems to indicate that apart from the follow-up time, also the post-injury phase influences the correspondence between directly reported and recalled scores. In the acute phase (1 week post-injury), there are rapid changes in health, which may impede recall, whereas the health state in the recovery phase (3 to 12 months post-injury) may be more comparable to the current health state and therefore easier to remember. These findings are interesting to study further in future studies, for example, to see how a 2-year time period affects the recalled outcomes.

Different subgroups of patients had a different degree of correspondence between the directly measured and recalled HRQL. As hypothesized, patients with a major trauma (ISS ≥ 16) had lower correspondence. This may be due to the severity of the trauma and possibly also due to neurologic complications many of them suffered from. The type and severity of injury thus also seem to influence the correspondence of recall. Also, patients with a middle level of education were among the groups with the lowest correspondence between directly measured and recalled, whereas correspondence was high among patients with a high level of education. To the best of our knowledge, no other studies have investigated whether the correspondence between directly measured and recalled HRQL is different among subgroups based on level of education.

Our finding that recalled EQ-5D-3L and EQ-VAS is systematically lower compared to the directly reported HRQL of trauma patients may have implications for the application of recalled EQ-5D in cost-effectiveness studies. The EQ-5D-3L is a widely applied HRQL instrument for QALY estimations and in cost-effectiveness analyses; however, systematic bias in retrospective assessment, resulting in larger differences in EQ-5D summary scores between two assessments compared to directly reported EQ-5D, can influence cost-effectiveness analyses, and therefore, use of recalled HRQL assessment can potentially lead to inefficiencies in resource allocation.

### Strengths and limitations

This study had several strengths and limitations. Strengths include the sample size of our study, which was large enough to test for differences between different subgroups of trauma patients, and the assessment of the directly reported and recalled HRQL on several time points and with different timeframes between assessments to evaluate both assessment points and follow-up times. Another strength is the inclusion of both the EQ-5D dimensions and the EQ-VAS, which allowed us to compare a classification-like scale with a more subjective scale. Limitations include the potential selection and participation bias and the use of the EQ-5D-3L instrument instead of the 5L version. A low proportion (< 10%) of all invited trauma patients participated in the study and filled in the various EQ-5D surveys at all assessment points. Therefore, our results may not fully reflect the Dutch trauma population. The EQ-5D-3L, the three answer option instrument, is less sensitive than the more comprehensive EQ-5D-5L version (five answer options). The recall correspondence is expected to be less accurate when more answer options are present. It might be valuable to test the recall correspondence of the EQ-5D-5L in future research.

## Conclusion

Our study showed that recalled HRQL measured by the EQ-5D-3L and EQ-VAS is systematically lower compared to the directly reported HRQL of trauma patients, with a general decrease over time. This indicates that recalled HRQL cannot be used as a replacement for prospectively assessed HRQL. If it is difficult or impossible to collect HRQL data prospectively, retrospective assessment is an option; however, when applying retrospective assessment, researchers should be aware that systematic bias may occur. Our study showed better correspondence for the EQ-VAS compared to the EQ-5D summary score, indicating that the EQ-5D descriptive system is more prone to systematic bias than EQ-VAS. Besides, patient characteristics, injury severity, subjectivity of the dimension, and time interval also influence correspondence between directly reported and recalled HRQL.

## Electronic supplementary material

Below is the link to the electronic supplementary material.

Supplementary material 1 (PDF 575 kb)
