# Supplementary material for: Correspondence of directly reported and recalled health-related quality of life in a large heterogeneous sample of trauma patients
Source: Qual Life Res. 2019 Jul 30;28(11):3005–13. doi: 10.1007/s11136-019-02256-z (PMC6803580; doi:10.1007/s11136-019-02256-z)
Supplement: Supplementary file 1 — Supplementary material 1 (PDF 575 kb) [file 11136_2019_2256_MOESM1_ESM.pdf]

Online Resource 1. Mean EQ-5D summary score assessed 1 week (T1) and recall at 3 months (T2) post-injury and intraclass correlation coefficients (ICC)

	n	EQ-5D assessed at T1 (1 week post-injury)	Recall EQ-5D assessed at T2 (3 months post-injury)	P-value	ICC (95% CI)
**Total**	446[$]	0.482 (SD 0.30)	0.453 (SD 0.34)	0.044*	0.575 (0.51, 0.63)
**Gender**					
Males	247	0.499 (SD 0.30)	0.491 (SD 0.35)	0.694	0.564 (0.47, 0.64)
Females	199	0.461 (SD 0.29)	0.406 (SD 0.33)	0.007*	0.586 (0.49, 0.67)
**Age**					
<65 years	231	0.457 (SD 0.29)	0.415 (SD 0.34)	0.049*	0.498 (0.40, 0.59)
65+ years	215	0.509 (SD 0.30)	0.494 (SD 0.34)	0.436	0.647 (0.56, 0.72)
**Educational level[1]**					
Low	97	0.495 (SD 0.34)	0.486 (SD 0.36)	0.791	0.609 (0.47, 0.72)
Middle	171	0.510 (SD 0.29)	0.437 (SD 0.36)	0.003*	0.527 (0.41, 0.63)
High	171	0.435 (SD 0.27)	0.443 (SD 0.32)	0.682	0.617 (0.52, 0.70)
**Comorbidity status[2]**					
No comorbidity	185	0.510 (SD 0.28)	0.475 (SD 0.33)	0.119	0.505 (0.39, 0.61)
Comorbidity	253	0.467 (SD 0.31)	0.443 (SD 0.35)	0.209	0.598 (0.51, 0.67)
**Injury Severity Score**					
ISS <16	417	0.490 (SD 0.30)	0.466 (SD 0.34)	0.086	0.573 (0.51, 0.64)
ISS >= 16	29	0.359 (SD 0.31)	0.278 (SD 0.38)	0.208	0.520 (0.20, 0.74)

SD=standard deviation, ICC=Intraclass correlation coefficient, ISS=injury severity score

[$] Patients who completed the EQ-5D at 1 week and recall of 1 week at 3 months and 12 months, and EQ-5D at 3 months and recall of 3 months at 12 months after sustaining an injury

[*] $p<0.05$

[1] 7 missing values, [2] 8 missing values

Online Resource 2. Mean EQ-5D summary score assessed 1 week (T1) and recall at 12 months (T3) post-injury and intraclass correlation coefficients (ICC)

	n	EQ-5D assessed at T1 (1 week post-injury)	Recall EQ-5D assessed at T3 (12 months post-injury)	P-value	ICC (95% CI)
**Total**	446[$]	0.482 (SD 0.30)	0.363 (SD 0.38)	<0.001*	0.518 (0.45, 0.58)
**Gender**					
Males	247	0.499 (SD 0.30)	0.407 (SD 0.39)	<0.001*	0.543 (0.45, 0.63)
Females	199	0.461 (SD 0.29)	0.309 (SD 0.36)	<0.001*	0.477 (0.36, 0.58)
**Age**					
<65 years	231	0.457 (SD 0.29)	0.307 (SD 0.37)	<0.001*	0.475 (0.37, 0.57)
65+ years	215	0.509 (SD 0.30)	0.423 (SD 0.38)	<0.001*	0.552 (0.45, 0.64)
**Educational level[1]**					
Low	97	0.495 (SD 0.34)	0.383 (SD 0.40)	0.001*	0.627 (0.49, 0.73)
Middle	171	0.510 (SD 0.29)	0.348 (SD 0.38)	<0.001*	0.423 (0.29, 0.54)
High	171	0.435 (SD 0.27)	0.358 (SD 0.37)	0.001*	0.548 (0.43, 0.64)
**Comorbidity status[2]**					
No comorbidity	185	0.510 (SD 0.27)	0.381 (SD 0.37)	<0.001*	0.526 (0.41, 0.62)
Comorbidity	253	0.467 (SD 0.31)	0.349 (SD 0.38)	<0.001*	0.519 (0.42, 0.60)
**Injury Severity Score**					
ISS <16	417	0.490 (SD 0.30)	0.380 (SD 0.38)	<0.001*	0.516 (0.44, 0.58)
ISS >= 16	29	0.359 (SD 0.31)	0.118 (SD 0.36)	0.015*	0.439 (0.09, 0.69)

SD=standard deviation, ICC=Intraclass correlation coefficient, ISS=injury severity score

[$] Patients who completed the EQ-5D at 1 week and recall of 1 week at 3 months and 12 months, and EQ-5D at 3 months and recall of 3 months at 12 months after sustaining an injury

*p<0.05

[1]7 missing values, [2]8 missing values

Online Resource 3. Mean EQ-5D summary score assessed 3 months (T2) and recall at 12 months (T3) post-injury and intraclass correlation coefficients (ICC)

	n	EQ-5D assessed at T2 (3 months post-injury)	Recall EQ-5D assessed at T3 (12 months post-injury)	P-value	ICC (95% CI)
**Total**	446[$]	0.737 (SD 0.24)	0.713 (SD 0.27)	0.022*	0.598 (0.54, 0.65)
**Gender**					
Males	247	0.744 (SD 0.23)	0.727 (SD 0.27)	0.233	0.584 (0.50, 0.66)
Females	199	0.729 (SD 0.24)	0.696 (SD 0.26)	0.034*	0.614 (0.52, 0.69)
**Age**					
<65 years	231	0.743 (SD 0.23)	0.704 (SD 0.27)	0.006*	0.631 (0.55, 0.70)
65+ years	215	0.731 (SD 0.24)	0.723 (SD 0.26)	0.611	0.564 (0.47, 0.65)
**Educational level[1]**					
Low	97	0.690 (SD 0.26)	0.699 (SD 0.28)	0.684	0.671 (0.55, 0.77)
Middle	171	0.742 (SD 0.23)	0.697 (SD 0.28)	0.024*	0.483 (0.36, 0.59)
High	171	0.757 (SD 0.22)	0.734 (SD 0.24)	0.115	0.673 (0.58, 0.75)
**Comorbidity status[2]**					
No comorbidity	185	0.784 (SD 0.23)	0.778 (SD 0.22)	0.634	0.644 (0.55, 0.72)
Comorbidity	253	0.705 (SD 0.23)	0.666 (SD 0.28)	0.012*	0.547 (0.46, 0.63)
**Injury Severity Score**					
ISS <16	417	0.742 (SD 0.23)	0.724 (SD 0.26)	0.097	0.594 (0.53, 0.65)
ISS >= 16	29	0.672 (SD 0.28)	0.556 (SD 0.30)	0.019*	0.614 (0.33, 0.80)

SD=standard deviation, ICC=Intraclass correlation coefficient, ISS=injury severity score

[$]Patients who completed the EQ-5D at 1 week and recall of 1 week at 3 months and 12 months, and EQ-5D at 3 months and recall of 3 months at 12 months after sustaining an injury

[*]$p<0.05$

[1]7 missing values, [2]8 missing values

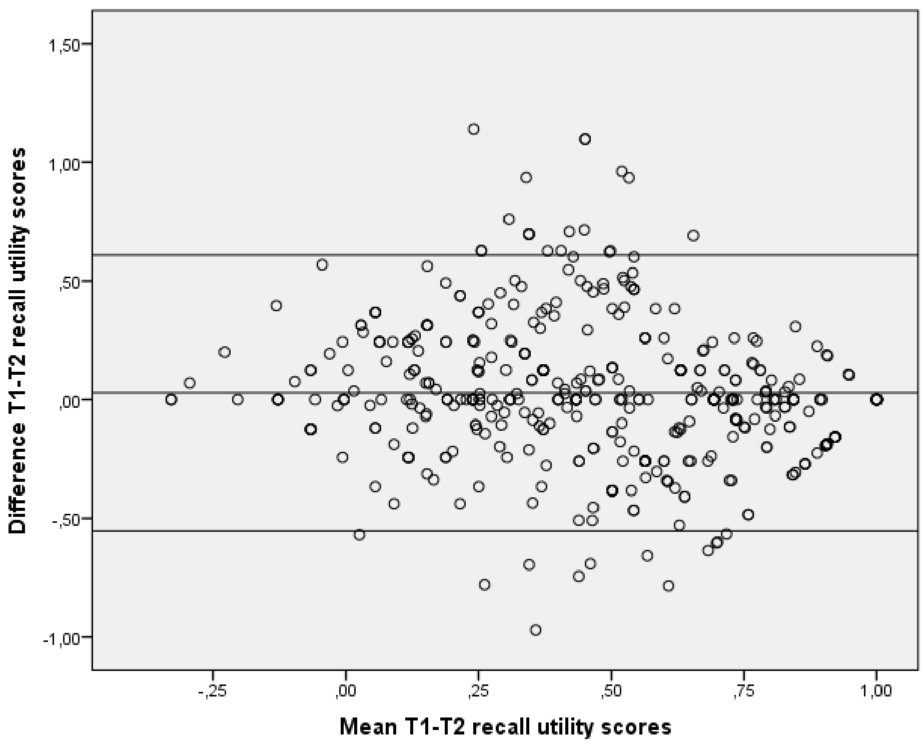

Online Resource 4. Bland-Altman plot for agreement T1 and T2 recall EQ-5D summary scores

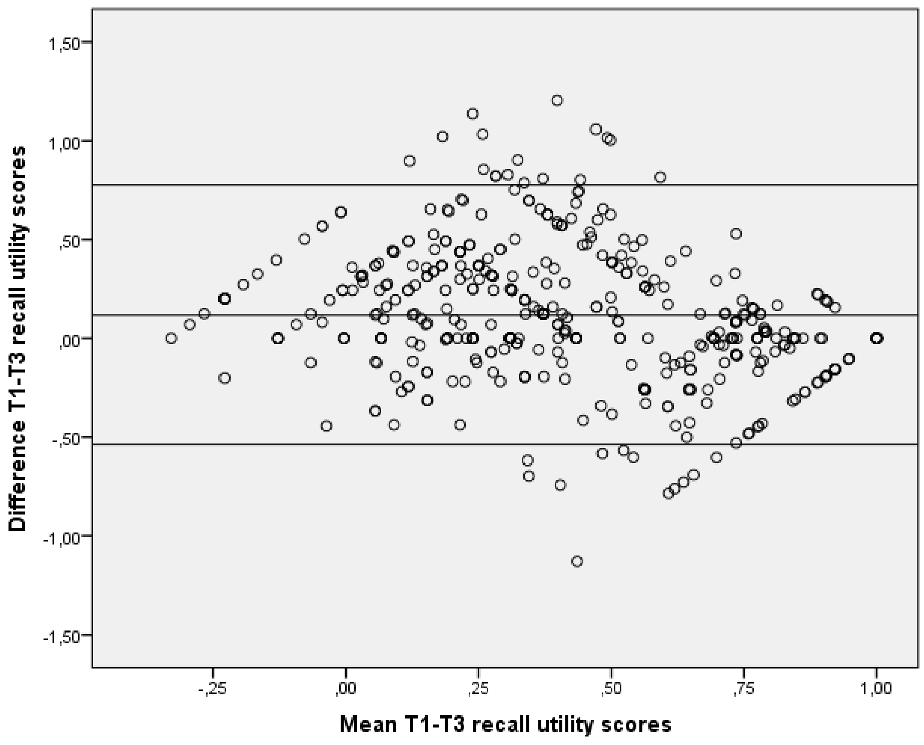

Online Resource 5. Bland-Altman plot for agreement T1 and T3 recall EQ-5D summary scores

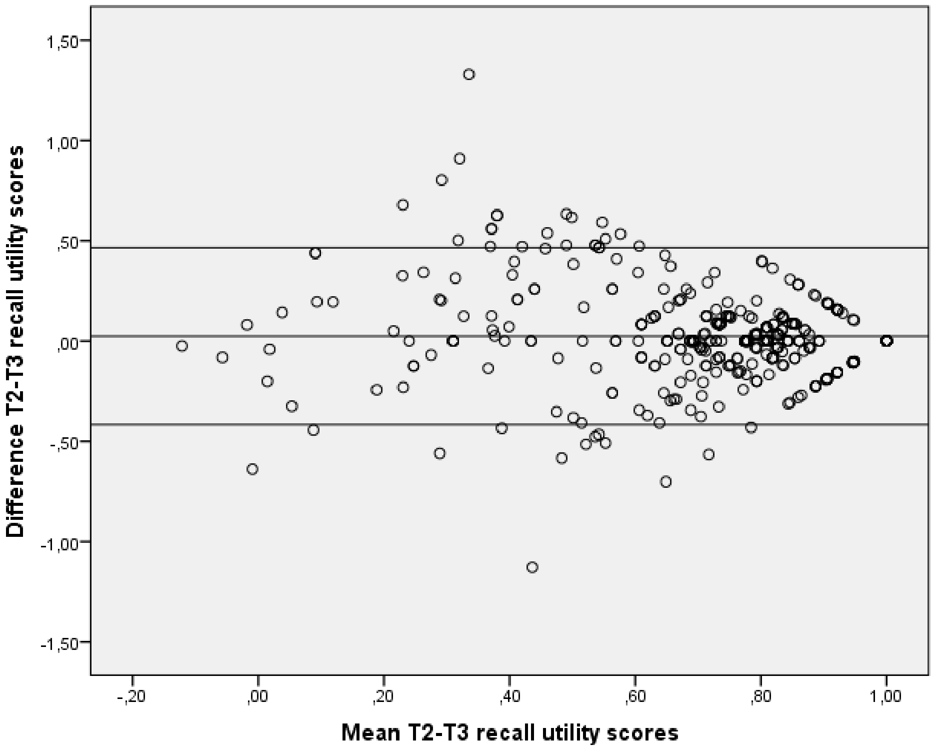

Online Resource 6. Bland-Altman plot for agreement T2 and T3 recall EQ-5D summary scores

Online Resource 7. Mean VAS scores assessed 1 week (T1) and recall at 3 months (T2) post-injury and intraclass correlation coefficients (ICC)

	n	VAS assessed at T1 (1 week post-injury)	VAS assessed at T2 (3 months post-injury)	P-value	ICC
**Total**	446[$]	56.3 (SD 20)	55.4 (SD 21)	0.304	0.578 (0.51, 0.64)
**Gender**					
Males	247	58.0 (SD 21)	56.7 (SD 22)	0.307	0.539 (0.44, 0.62)
Females	199	54.1 (SD 19)	53.7 (SD 19)	0.743	0.636 (0.55, 0.71)
**Age**					
<65 years	231	53.9 (SD 20)	50.7 (SD 21)	0.007*	0.614 (0.53, 0.69)
65+ years	215	58.9 (SD 20)	60.4 (SD 20)	0.242	0.517 (0.41, 0.61)
**Educational level[1]**					
Low	97	55.2 (SD 20)	56.2 (SD 21)	0.643	0.546 (0.39, 0.67)
Middle	171	58.2 (SD 20)	55.0 (SD 22)	0.038*	0.552 (0.44, 0.65)
High	171	54.7 (SD 20)	54.8 (SD 20)	0.952	0.621 (0.52, 0.71)
**Comorbidity status[2]**					
No comorbidity	185	57.7 (SD 19)	56.2 (SD 21)	0.273	0.548 (0.44, 0.64)
Comorbidity	253	55.4 (SD 20)	55.3 (SD 21)	0.925	0.568 (0.48, 0.65)
**Injury Severity Score**					
ISS <16	417	57.2 (SD 20)	56.4 (SD 20)	0.374	0.597 (0.53, 0.66)
ISS >= 16	29	42.9 (SD 22)	40.0 (SD 24)	0.603	0.188 (-0.19, 0.51)

SD=standard deviation, ICC=Intraclass correlation coefficient, ISS=injury severity score

[$] Patients who completed the EQ-5D at 1 week and recall of 1 week at 3 months and 12 months, and EQ-5D at 3 months and recall of 3 months at 12 months after sustaining an injury

[*] $p<0.05$

[1] 7 missing values, [2] 8 missing values

Online Resource 8. Mean VAS scores assessed 1 week (T1) and recall at 12 months (T3) post-injury and intraclass correlation coefficients (ICC)

	n	VAS assessed at T1 (1 week post-injury)	VAS assessed at T3 (12 months post-injury)	P-value	ICC
**Total**	446[$]	56.3 (SD 20)	53.3 (SD 23)	0.002*	0.561 (0.49, 0.62)
**Gender**					
Males	247	58.0 (SD 21)	54.7 (SD 24)	0.016*	0.544 (0.45, 0.63)
Females	199	54.1 (SD 19)	51.4 (SD 22)	0.046*	0.579 (0.48, 0.66)
**Age**					
<65 years	231	53.9 (SD 20)	47.8 (SD 22)	<0.001*	0.555 (0.46, 0.64)
65+ years	215	58.9 (SD 20)	59.1 (SD 23)	0.860	0.552 (0.45, 0.64)
**Educational level[1]**					
Low	97	55.2 (SD 20)	54.4 (SD 25)	0.658	0.647 (0.52, 0.75)
Middle	171	58.2 (SD 20)	51.8 (SD 24)	<0.001*	0.434 (0.60, 0.55)
High	171	54.7 (SD 20)	53.8 (SD 21)	0.491	0.652 (0.56, 0.73)
**Comorbidity status[2]**					
No comorbidity	185	57.7 (SD 19)	55.4 (SD 23)	0.150	0.503 (0.39, 0.60)
Comorbidity	253	55.4 (SD 20)	51.8 (SD 23)	0.004*	0.576 (0.49, 0.65)
**Injury Severity Score**					
ISS <16	417	57.2 (SD 20)	54.6 (SD 22)	0.007*	0.564 (0.50, 0.63)
ISS >= 16	29	42.9 (SD 22)	33.9 (SD 23)	0.082	0.292 (-0.08, 0.59)

SD=standard deviation, ICC=Intraclass correlation coefficient, ISS=injury severity score

[$] Patients who completed the EQ-5D at 1 week and recall of 1 week at 3 months and 12 months, and EQ-5D at 3 months and recall of 3 months at 12 months after sustaining an injury

[*] $p<0.05$

[1] 7 missing values, [2] 8 missing values

Online Resource 9. Mean VAS scores assessed 3 months (T2) and recall at 12 months (T3) post-injury and intraclass correlation coefficients (ICC)

	n	VAS assessed at T2 (3 months post-injury)	VAS assessed at T3 (12 months post-injury)	P-value	ICC
**Total**	446[$]	72.5 (SD 17)	68.0 (SD 17)	<0.001*	0.595 (0.53, 0.65)
**Gender**					
Males	247	73.0 (SD 17)	68.7 (SD 18)	<0.001*	0.642 (0.56, 0.71)
Females	199	71.9 (SD 18)	67.1 (SD 17)	<0.001*	0.535 (0.43, 0.63)
**Age**					
<65 years	231	71.8 (SD 18)	65.2 (SD 18)	<0.001*	0.581 (0.49, 0.66)
65+ years	215	73.3 (SD 16)	71.0 (SD 17)	0.020*	0.618 (0.53, 0.69)
**Educational level[1]**					
Low	97	69.6 (SD 17)	67.2 (SD 19)	0.166	0.561 (0.41, 0.68)
Middle	171	74.2 (SD 17)	67.9 (SD 18)	<0.001*	0.512 (0.39, 0.62)
High	171	72.6 (SD 16)	68.6 (SD 17)	<0.001*	0.703 (0.62, 0.77)
**Comorbidity status[2]**					
No comorbidity	185	76.6 (SD 17)	72.0 (SD 16)	<0.001*	0.627 (0.53, 0.71)
Comorbidity	253	69.9 (SD 16)	65.1 (SD 18)	<0.001*	0.551 (0.46, 0.63)
**Injury Severity Score**					
ISS <16	417	72.9 (SD 17)	68.8 (SD 17)	<0.001*	0.585 (0.52, 0.65)
ISS >= 16	29	66.5 (SD 19)	56.6 (SD 18)	0.002*	0.657 (0.39, 0.82)

SD=standard deviation, ICC=Intraclass correlation coefficient, ISS=injury severity score
[$] Patients who completed the EQ-5D at 1 week and recall of 1 week at 3 months and 12 months, and EQ-5D at 3 months and recall of 3 months at 12 months after sustaining an injury
[*] $p<0.05$
[1] 7 missing values, [2] 8 missing values

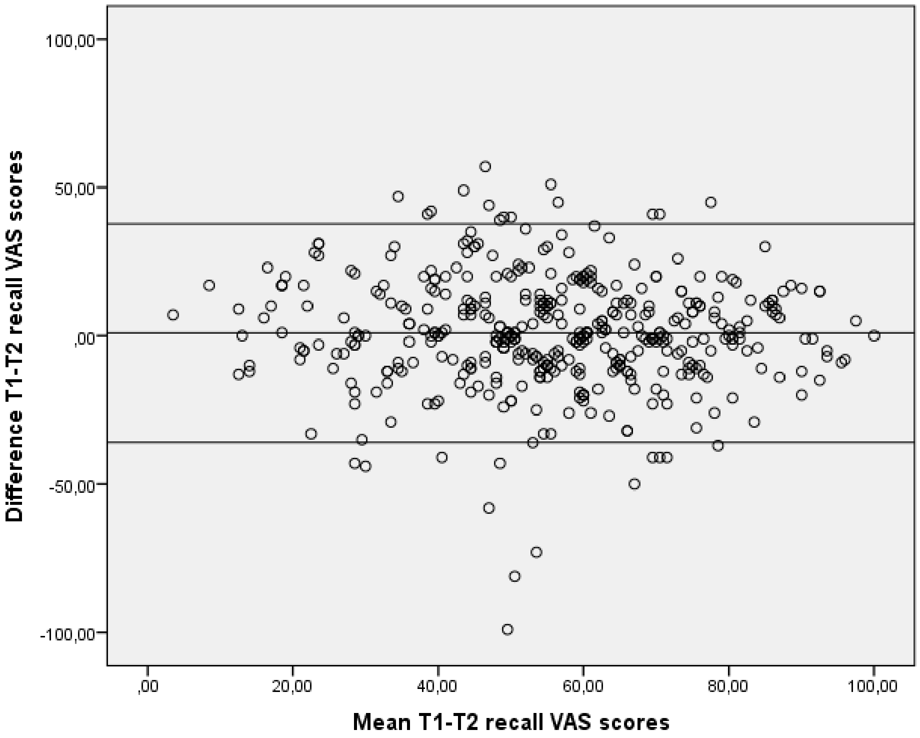

Online Resource 10. Bland-Altman plot for agreement T1 and T2 recall EQ-VAS scores

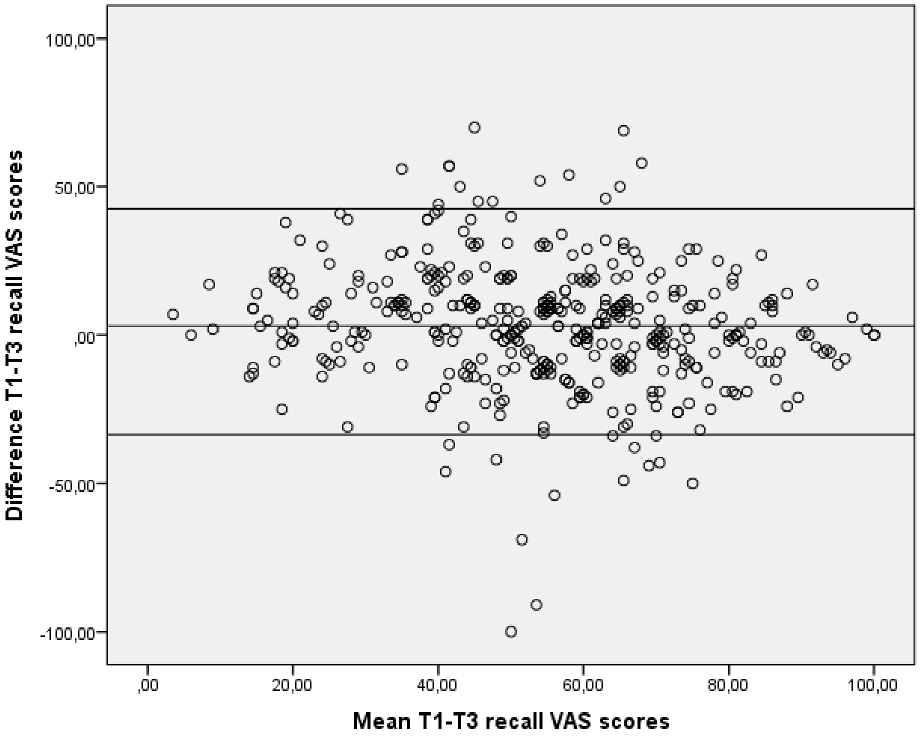

Online Resource 11. Bland-Altman plot for agreement T1 and T3 recall EQ-VAS scores

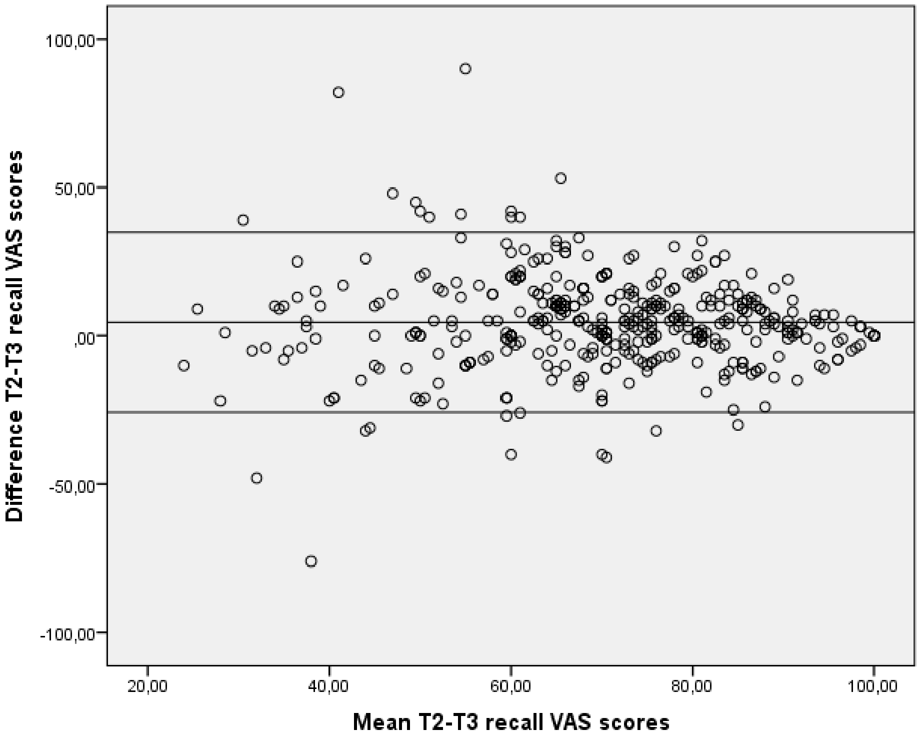

Online Resource 12. Bland-Altman plot for agreement T2 and T3 recall EQ-VAS scores
